# Role of Resveratrol in Prevention and Control of Cardiovascular Disorders and Cardiovascular Complications Related to COVID-19 Disease: Mode of Action and Approaches Explored to Increase Its Bioavailability

**DOI:** 10.3390/molecules26102834

**Published:** 2021-05-11

**Authors:** Nikola Gligorijević, Dragana Stanić-Vučinić, Mirjana Radomirović, Marija Stojadinović, Urmila Khulal, Olgica Nedić, Tanja Ćirković Veličković

**Affiliations:** 1 Institute for the Application of Nuclear Energy, Department for Metabolism, University of Belgrade, Banatska 31b, 11080 Belgrade, Serbia; nikolag@inep.co.rs (N.G.); olgica@inep.co.rs (O.N.); 2 Center of Excellence for Molecular Food Sciences, Department of Biochemistry, Faculty of Chemistry, University of Belgrade, Studentski trg 12–16, 11000 Belgrade, Serbia; dstanic@chem.bg.ac.rs (D.S.-V.); radomirovicmirjana@chem.bg.ac.rs (M.R.); mstojadinovic@chem.bg.ac.rs (M.S.); 3 Faculty of Bioscience Engineering, Ghent University, 9000 Ghent, Belgium; Urmila.Khulal@ghent.ac.kr; 4 Global Campus, Ghent University, Yeonsu-gu, Incheon 406-840, Korea; 5 Serbian Academy of Sciences and Arts, Knez Mihailova 35, 11000 Belgrade, Serbia

**Keywords:** resveratrol, cardiovascular disease, bioavailability, diet, COVID-19, resveratrol carriers, cardiovascular protection

## Abstract

Resveratrol is a phytoalexin produced by many plants as a defense mechanism against stress-inducing conditions. The richest dietary sources of resveratrol are berries and grapes, their juices and wines. Good bioavailability of resveratrol is not reflected in its high biological activity in vivo because of resveratrol isomerization and its poor solubility in aqueous solutions. Proteins, cyclodextrins and nanomaterials have been explored as innovative delivery vehicles for resveratrol to overcome this limitation. Numerous in vitro and in vivo studies demonstrated beneficial effects of resveratrol in cardiovascular diseases (CVD). Main beneficial effects of resveratrol intake are cardioprotective, anti-hypertensive, vasodilatory, anti-diabetic, and improvement of lipid status. As resveratrol can alleviate the numerous factors associated with CVD, it has potential as a functional supplement to reduce COVID-19 illness severity in patients displaying poor prognosis due to cardio-vascular complications. Resveratrol was shown to mitigate the major pathways involved in the pathogenesis of SARS-CoV-2 including regulation of the renin-angiotensin system and expression of angiotensin-converting enzyme 2, stimulation of immune system and downregulation of pro-inflammatory cytokine release. Therefore, several studies already have anticipated potential implementation of resveratrol in COVID-19 treatment. Regular intake of a resveratrol rich diet, or resveratrol-based complementary medicaments, may contribute to a healthier cardio-vascular system, prevention and control of CVD, including COVID-19 disease related complications of CVD.

## 1. Introduction

### 1.1. Structure of Resveratrol

Resveratrol (trans-3,5,4′-trihydroxystilbene), a stilbene phenolic compound, was first isolated from the root of white hellebore (*Veratrum grandiflorum* O. Loes) in 1940, and detected in *Vitis vinifera* grapevines in 1976 [1]. Discovery of its presence in red wine in 1992 was used to explain “French Paradox”, assumed to be responsible for low rates of coronary heart disease mortality in some areas of France, despite the elevated intake of fat and cholesterol through the daily diet [2]. Resveratrol is also an important constituent of the ayurvedic herbal tonic ‘drakchasava’ made from grapes and prescribed as a cardiotonic [3].

**Figure 1:**
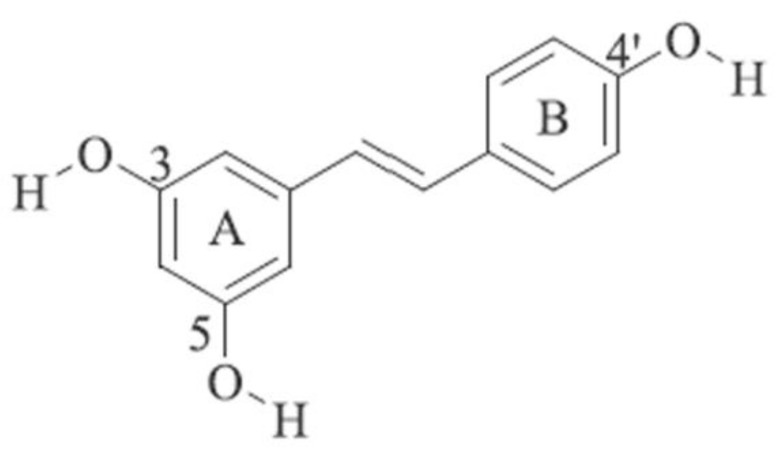
Molecular structure of resveratrol.

Resveratrol is a compound with two aromatic rings linked by an ethylene bridge (molecular weight of 228.247 g/mol), ring A has two hydroxyl groups at C3 and C5, and ring B has one hydroxyl group at C4′ (Figure 1). Due to the presence of the central ethylene moiety in its structure, resveratrol has two possible stereoisomers, *cis* and *trans*. The naturally occurring resveratrol is usually its *trans*-isomer (E-configuration) and the majority of reported health benefits are attributed to this form. When exposed to UV and visible light, *trans*-resveratrol converts to *cis*-resveratrol (Z) by photo-isomerization [4]. *Cis*-resveratrol is not well explored despite the findings from in vivo intestinal epithelial model studies that *trans*-resveratrol converts to *cis*-resveratrol in vivo [5] and that *cis*-resveratrol is also a biologically active form [6]. The glucose-bound form of resveratrol, piceid (also named Polydatin), is the major resveratrol derivative in food sources and it is converted to *trans*-resveratrol by hydrolysis. As a result of its hydroxyl groups and double C-C bond, resveratrol is sensitive to light, oxygen, alkaline pH and increased temperature [7].

Resveratrol oligomers (ROs) can be found in several plants. They are biosynthesized by the successive condensation of resveratrol units and their structural diversity originates from patterns of phenoxy radical–radical coupling. This phenomenon yields various fused-ring systems containing asymmetric carbons, resulting in regioisomerism and stereoisomerism [8].

Hydroxyl groups, aromatic rings and a double bond enable resveratrol to be modified into derivatives with higher bioactivity as well as diversified therapeutic efficacies. In the last several years, a series of resveratrol derivatives has been synthesized and their bioactivities have been evaluated [9]. Structure–activity relationship (SAR) studies indicate that the bioactivity and the stability of resveratrol might be improved after modification of its hydroxyl groups, benzene rings and double C-C bond leading to the formation of resveratrol derivatives [10]. Methoxylated, hydroxylated and halogenated derivatives of resveratrol were particularly recognized for their diverse therapeutic potential [9]. Although increasing the number of −OH groups at their ortho position on the stilbene phenol ring could increase the antioxidant and cytotoxic activity, it also leads to fast metabolism and weak bioavailability [11]. The O-methylation in stilbene ring improves stability of these compounds as well as increases the lipophilicity and thus the cell uptake [12]. Throughout recent decades, medicinal chemists have synthesized a lot of novel derivatives and analogues of resveratrol using different modification strategies to improve its pharmacokinetic properties and its biological activity, such as anticarcinogenic activity [13].

### 1.2. Occurrence in Nature

Resveratrol is a phytoalexin produced by many plants in response to UV radiations, mechanical injury or attack by pathogens (such as bacteria and fungi) as a defense mechanism against these stress-inducing conditions. It is commonly found in about 100 plant species of 34 families including Pinaceae, Moraceae, Liliaceae, Polygonaceae, Vitaceae and Leguminoseae [10]. It is accumulated in skins, roots, stems, leaves, flowers and seeds due to environmental stimulation. In general, stilbenes are less common plant phenolics and the richest dietary sources of resveratrol are berries and grapes, their juices and wines. Abundance of resveratrol in different sources is presented in Table 1.

Food processing byproducts are considered readily available economical sources of resveratrol, such as grape pomace, cane and leaves [14], peanut roots [15], soybean seed coat [16], hop pellets [17] and so on. For instance, grape cane contains about 5 mg/g dry weight [18]. Besides, non-dietary alternative resveratrol sources are also investigated, such as black spruce bark [19], Japanese knotweed roots [20] and tree peony seed coats [21]. In addition to resveratrol extraction from natural sources, chemical synthesis/biosynthesis of resveratrol is also prevalent via Heck reaction, Perkin reaction, Wittig reaction and biomimetic synthesis [10]. The yield of resveratrol extracted from plants or chemical synthesis is relatively low; therefore, plant in vitro culture [22], microbial [23] and biocatalytic [24] approaches are alternatively implemented for higher production yield of resveratrol and its derivatives.

### 1.3. Supplementation of Diet with Resveratrol

Resveratrol supplementation may be an effective method to make this bioactive compound accessible to consumers. Safe wine drinking (500 mL/day) could provide few mg of resveratrol daily while one supplement capsule could provide more than 500 mg of pure *trans*-resveratrol. In one of the survey studies to predict the attitude and adoption intentions of resveratrol consumers, the potential consumers were identified by their usage of complementary and alternative medicine, rather than their healthy behaviors [25].

Although there is numerous literature data on the protective effects of resveratrol against diseases, clinical studies on resveratrol toxicity and adverse effects are relatively scarce. However, many studies indicate harmful effects of resveratrol, and potential adverse effects of resveratrol are recently comprehensively reviewed, based on summarized molecular evidences, as well as in vitro and in vivo non-human and human evidences [26]. As resveratrol may inhibit cytochrome P450 3A4 (CYP3A4) activity, the high intake of the supplements could potentially reduce metabolic clearance of drugs that undergo extensive first-pass CYP3A4 metabolism simultaneously increasing the bioavailability and toxicity risk of these drugs [27]. Also, when taken with anticoagulant and antiplatelet drugs, it could enhance both bruising and bleeding risk due to resveratrol ability to hinder human platelet aggregation in vitro [26]. On the other hand, it is speculated that higher resveratrol doses could compete with other polyphenols for transporters, reducing both their uptake and potential synergistic effects [28].

Although resveratrol is safe and reasonably tolerated at up to 5 g/day taken for a month [29], side effects such as nausea, vomiting, diarrhea and liver dysfunction in patients with non-alcoholic fatty liver disease were recorded at doses of more than 2.5 g/day [30]. The specific resveratrol doses used in various clinical trials differ dramatically from 10 mg/day to 5 g/day [31,32] showing an effect on insulin sensitivity [33] and increased flow-mediated dilation [34]. The upper limit of resveratrol dose for clinical trials has been suggested as low as 1 g/day because of the observed minimal adverse reactions and metabolic interactions with cytochrome P-450 complex enzymes at this dose [30]. However, there is still no consensus regarding the usage of resveratrol based on scientific evidence. The studies indicate a biphasic dose-response of resveratrol wherein the low doses of resveratrol have health-promoting effects while higher dose treatments reveal detrimental effects. This hormetic dose-response is characterized by low-dose stimulation and a high-dose inhibition [6].

Most supplements in the form of capsules contain 250 to 500 milligrams per single dose, which is lower than the amounts that have shown health benefits in most of the research. As with other forms of supplements, there is no recommended resveratrol dosage.

## 2. Bioavailability of Resveratrol

### 2.1. Intake and Metabolism

The oral absorption of resveratrol is about 75% while the remaining 25% is eliminated via excretion [35]. It rapidly undergoes extensive transformation in the gastrointestinal tract and liver via sulfation and glucuronidation. Once absorbed into the enterocyte, resveratrol undergoes sulfation by SULT1A1 and glucuronidation by UGT1A1 and UGTA9 (phase II biotransformation) resulting in 3′-O-β-d-glucuronide, 4′-O-d-glucuronide and 3-O-sulfate forms. The conjugated resveratrol exits enterocyte on the apical membrane via BRCP and MRP2 transporters and is metabolized by the gut microbiota to generate dihydroresveratrol, 3,40-dihydroxy-trans-stilbene and 3,40-dihydroxybibenzyl in the large intestine. A small fraction of resveratrol escapes conjugation and exits the enterocyte via the basolateral membrane. Resveratrol and metabolites that exit the enterocyte on the basolateral membrane (via MRP3 transporter) enter portal circulation and in the liver, SULT1A1, UGT1A1 and UGTA9 further conjugate resveratrol. From the liver, resveratrol metabolites enter systemic circulation and are absorbed by peripheral tissues. Resveratrol metabolites also undergo enterohepatic circulation, being reabsorbed in the intestine after hydrolysis. It is further metabolized in the liver after entering the portal circulation [36].

Therefore, the concentration of resveratrol metabolites in plasma is much higher than the concentration of untransformed resveratrol [37]. In a study of oral resveratrol intake of 5 mg and 1000 mg by healthy volunteers, total plasma [14C]-resveratrol equivalents increased linearly reaching average peak concentrations of 0.6 and 137 μmol/L respectively. At both doses, maximal plasma concentrations were observed around the 1h time point and [14C]-labelled species were still detectable after 24 h of resveratrol intake [38].

More than 20 resveratrol metabolites have been found in human plasma, urine and some tissues including its *trans*- and/or *cis*- forms of mono- and diglucuronides, mono-, di- and trisulfates and sulfoglucuronides as well as equivalent conjugations of the microbiota-derived metabolites [39]. The sulfated conjugates partly maintain their bioactivity but it seems that their activity decreases as the degree of sulfation increases [40]. Resveratrol-3-O-sulfate was found to exert pronounced anti-estrogen activity [41], and anticarcinogenic [42,43] and cardioprotective [44] effects. In humans, the most abundant circulating resveratrol metabolite was found to be 3-O-sulfate with maximum reported Cmax of about 18 μM after intake of 5 g resveratrol while Cmax of parent resveratrol was only about 4.2 μM [30].

When resveratrol is compared to pterostilbene, a single hydroxyl group in pterostilbene makes it a less favorable substrate of glucuronidase and sulfatase resulting in a better metabolic stability of pterostilbene than resveratrol [39]. There has been approach for production of more potent resveratrol metabolites such as, piceatannol (3,40,30,5-tetrahydroxistilbene), a C-30 hydroxylated product [45].

### 2.2. Tissue Bioavailability

During the past few years, an increasing number of human clinical trials have been carried out to study resveratrol biological activity besides conventional in vitro and animal model systems. According to database ClinicalTrials.gov, there are currently 166 clinical trials investigating the health effects of resveratrol. The clinical benefits of resveratrol are still not adequately well-defined, mainly due to its unfavorable pharmacokinetics/pharmacodynamics profile, poor bioavailability, low aqueous solubility, chemical instability and rapid clearance from systemic circulation [13].

The biological activity of resveratrol is limited by isomerization into the *cis*-form and its poor solubility in aqueous solutions resulting in biological permeation [46]. Additionally, resveratrol is sensitive to alkaline pH, temperature fluctuation and exposure to light and oxygen [7,47]. Therefore, there have been numerous researches in attempt to increase resveratrol stability, solubility and permeation in biological membranes enabling its controlled release rate and targeted delivery in the last few years. Several drug delivery systems were investigated including cubosomes, cyclodextrins, dendrimers, liquid crystals, liposomes, micelles, microspheres, nano- and microemulsions, lipid nanocarriers and polymeric films [46]. Besides, there are resveratrol analogs with bioactivities similar to resveratrol with increased oral bioavailability such as pterostibene [48] and α-methylstilbene [49].

Resveratrol tissue bioavailability is still controversial, and plasma concentration of its metabolites determines the exact resveratrol bioavailability and its amount available to peripheral tissues. The studies based on animal models and human clinical trials point the gastrointestinal tract as the main target of resveratrol distribution and its derivatives. In pigs, 65% of resveratrol and its metabolites were found in the gastrointestinal tract, 7.7% in urine, 1.2% in bile and only 0.5% in organs 6 h after intragastric administration of 472 mg resveratrol [50]. Similarly, following administration of a single 150 mg/kg dosage of *trans*-resveratrol-3-O-glucoside in rats, the digestive tract was found to be the major distribution tissue of resveratrol and its derivatives at 150 mg/kg dosage of, in contrast to which, resveratrol was found poorly distributed into the brain indicating their inability to cross the blood-brain barrier [51]. Human study indicates that the concentration of resveratrol equivalents is lower in the underlying muscle layer than in surface mucosal layer of colorectal tissue obtained from patients that received 1 g resveratrol daily for one week [38]. In humans, resveratrol and its metabolites were detected in the ocular tissues after oral administration of three doses of one capsule daily of Longevinex (Resveratrol Partners LLC, Las Vegas, NV, USA), containing 100 mg of *trans*-resveratrol [52].

In mice, after gavage dosing of resveratrol 20 mg/kg, observed regeneration of free resveratrol (about 2%) into circulation suggested in vivo hydrolysis of its conjugates [53]. This is in agreement with the observation that human aortic endothelial cell lines are able to deconjugate resveratrol metabolites to free resveratrol when exposed to 0.5–10 μM *trans*-resveratrol during 16 h, suggesting their role as a reservoir for resveratrol regeneration [54]. These studies also demonstrate that the metabolites contribute to the intracellular concentration and activity of resveratrol.

While absorption of resveratrol from dietary sources (wine, grape and vegetable juice), when administrated at 25 mg/70 kg in various matrices, is similar to that from the supplements [55], its bioavailability from wine and grape juice, at about 14 μg/kg, was at least six-fold higher than that from tablets, implying matrix effect on its bioavailability and its enhancement by other constituents present in natural sources [56]. In order to bypass the gastrointestinal tract completely, few trials have investigated potential delivery routes beyond oral administration. Intravenous administration of 2, 10 and 20 mg/kg *trans*-resveratrol, *trans*-resveratrol glucuronide and *trans*-resveratrol sulfate in rats resulted in higher level of resveratrol plasma concentration compared to oral administration [57]. Plasma concentration of about 1.4 μM was achieved in two healthy humans following administration of a 140 mg buccal dosage of resveratrol [58].

### 2.3. Overcoming Low Bioavailability of Resveratrol—Available Approaches

Positive effects of resveratrol revealed by in vitro studies are not correlative with in vivo studies, probably owing to its inefficient systemic delivery. Indeed, low amounts of intact resveratrol were found in systemic circulation in human pharmacokinetic studies [37]. Limited bioavailability of resveratrol arises from its poor water solubility, low stability against heat- and light-induced oxidation as well as its high hepatic uptake [59]. Resveratrol is effectively absorbed by the intestine; however, its concentration in circulation drops below 1% due to its rapid metabolism in the liver and intestine (first pass effect) [60]. Studies performed on rats have shown that at least 30% of administered resveratrol is excreted via feces and urine in the form of conjugated metabolites [61]. Also, *trans*-resveratrol is sensitive to light; it changes conformation to the not as biologically active *cis* form upon exposure [28].

Different methods are being explored to improve resveratrol’s bioavailability and thus take advantage of its therapeutic potential; for instance, co-administration of resveratrol with inhibitors of glucuronidation [62,63], use of resveratrol methylated polyphenol analogs with better pharmacokinetic properties [64] and design of new delivery systems. This section will discuss some ways of overcoming issues of resveratrol delivery, including consequences of its interactions with proteins from food matrix and circulation, interactions with cyclodextrins and the usage of nanomaterials as carriers of resveratrol.

#### 2.3.1. Proteins as Carriers for Resveratrol Delivery

Due to its low water solubility, resveratrol needs to bind with other molecules to maintain its solubility as much as possible. The proteins identified as binding partners of resveratrol are serum albumins, hemoglobin and fibrinogen from circulation as well as proteins used in the food industry like β-lactoglobulin, buttermilk proteins, zein and gliadin.

Both human serum albumin (HSA) [65] and bovine serum albumin (BSA) [66] are shown to bind resveratrol with moderate affinity. When bound to resveratrol, the structure of both HSA and BSA remains almost unaltered. At higher concentration, resveratrol stabilizes HSA [65]. An important aspect of the HSA-resveratrol complex is that HSA can protect resveratrol from degradation, particularly from harmful effects of UV radiation. It is demonstrated that the limited solvent contact and the protein matrix effect limiting the freedom of fluctuation are responsible for the stabilization of *trans*-resveratrol molecules [67]. Hemoglobin is also a resveratrol binding protein, although with a lower affinity than HSA [68]. Both HSA and hemoglobin play an important role in the blood transport of resveratrol considering the high concentrations of these proteins in circulation.

Human fibrinogen is also shown to bind and protect resveratrol [69] and mutual oxidative protection of both partners was observed in the complex of resveratrol bound to fibrinogen. Therefore, fibrinogen may prolong the activity of resveratrol and release it unaltered from the complex at the site of injury thus enabling it to exert a positive effect on a healing process. Since the fibrinogen increases the solubility of resveratrol in aqueous environments (due to complex formation), interaction with this protein might increase the bioavailability of resveratrol. Experiments have also shown that resveratrol has no effect on fibrin formation and fibrinolysis [70] confirming that its intake is beneficial with respect to fibrinogen function.

An interesting study showed that resveratrol and its metabolites are present in human low-density lipoprotein (LDL) particles after moderate wine consumption (250 mL). These results indicate that LDL particles represent another way of resveratrol transportation in circulation. The presence of resveratrol and its metabolites in LDL can protect them from oxidation, which is an important step in atherosclerosis development. Resveratrol metabolites found in LDL particles are *trans*-resveratrol-3-O-glucuronide, *cis*-resveratrol-3-O-glucuronide, *cis*-resveratrol-3-O-glucoside, free *trans*-resveratrol, resveratrol-4′-O-glucuronide and *trans*-resveratrol-4′-O-glucoside [71].

Similarly, whole buttermilk was shown to bind resveratrol. A major resveratrol interaction was revealed with the buttermilk protein components, which increased its water solubility, making whole buttermilk a useful food carrier for resveratrol [72]. β-lactoglobulin, a major whey protein, binds resveratrol with moderate-high affinity. This interaction not only significantly increases resveratrol solubility but also protects against UV-induced degradation. The secondary structure of resveratrol-bound β-lactoglobulin remains intact, however, some alterations are observed in its tertiary structure [73]. Both α- and β-casein also bind resveratrol and may act as in vitro carriers of resveratrol [74,75]. It seems that buttermilk proteins are good resveratrol carriers as they enhance both the stability and water solubility of this polyphenol.

For resveratrol encapsulation, both zein and gliadin, water-insoluble proteins, were tested. Interestingly, zein interacts with resveratrol mainly through hydrogen bonds while gliadin binds resveratrol via hydrophobic interactions. Also, binding affinity increased for the gliadin-resveratrol pair and decreased for the zein-resveratrol pair at higher temperatures. This could be important when designing carrier systems for temperature-controlled retention or release of resveratrol [76]. Glycosylated gliadin has increased water solubility and it binds resveratrol with higher affinity compared to non-modified protein. The water solubility of resveratrol significantly increased when bound to glycosylated gliadin, making it an effective carrier for resveratrol [77].

#### 2.3.2. Application of Cyclodextrins in Resveratrol Delivery

Besides proteins, cyclodextrins (CD) are also potentially applicable carriers for resveratrol. Cyclodextrins are cyclic oligomers of glucopyranose units with a hydrophobic interior and hydrophilic exterior. The complex formation between resveratrol and sulfobutylether-β-CD significantly increased the water solubility of resveratrol while loading them into polymeric nanoparticles [78]. The resveratrol solubility improved due to its complexation with permethylated β-CD, 2,6-dimethylated β-CD and permethylated α-CD [79] and the photostability was achieved because of its complexation with carboxymethyl-β-CD [80]. By using a cyclodextrin glucanotransferase enzyme, resveratrol α-glycosides were synthesized directly from β-CD-resveratrol complexes in water. Major products formed were 3-O-α-d-glucosyl-resveratrol, 4′-O-α-d-glucosyl-resveratrol, 3-O-α-d-maltosyl-resveratrol and 4′-O-α-d-maltosyl-resveratrol. The increased water solubility of resveratrol was thus achieved while 4′−OH position on resveratrol is the best position for the glycosylation for maintaining its highest antiradical properties [81]. The resveratrol complexation with γ-CD in lemon juice enhanced its solubility and retained its antioxidant activity [82]. The antioxidant activity of *trans*-resveratrol was improved because of its complexation with (2-hydroxypropyl)-β-CD [83].

#### 2.3.3. Application of Nanomaterials in Resveratrol Delivery

Nanotechnology-based resveratrol delivery systems, as means of increasing its bioavailability and consequently its therapeutic potential, are lately gaining strong research interest. Various biocompatible materials of different working range sizes that are non-toxic, easily digested and metabolized, are being developed as potential drug delivery systems in addition to conventional oil-in-water nanoemulsion systems such as pickering emulsions, microbubbles, liposomes and gold nanoparticles [84,85].

Peanut oil, as an organic phase with a combination of lipophilic and hydrophilic emulsifiers, is a promising food-compatible delivery system for resveratrol. Resveratrol encapsulated in oil-in-water nanoemulsions of subcellular size based on soy lecithin/sugar esters and glycerol monooleate/Tween-20 retains the antioxidant activity of unencapsulated resveratrol, while at the same time preserving its stability upon high temperature and UV-light exposure [86]. Blank nanoemulsions, i.e., without resveratrol, have no negative effect on Caco-2 cell viability or the cytoskeleton structure. The rate of transport of nanoencapsulated resveratrol through the cell monolayers is higher than the rate of metabolization of emulsion droplets inside the cells, thus allowing protection of resveratrol molecules during intestinal transport. Sustained release of the encapsulated resveratrol enables resveratrol to reach its target sites in active form [87]. In line with this, encapsulation of resveratrol in the spontaneously formed nanoemulsions made of a mixture of grape seed oil and orange oil protected resveratrol against UV-light isomerization and degradation because of the emulsions with a droplet size closer to the wavelength of UV-light conferring better protection [88].

Biopolymer-based nanoparticles consisting of protein, polysaccharide and small molecule biosurfactant for delivery of resveratrol have also been fabricated. Tea saponin (TS) significantly improves resveratrol loading efficiency, photostability and thermal stability in the zein-propylene glycol alginate (PGA)-TS complex nanoparticles and at the same time facilitates the delayed release of resveratrol from the zein-PGA-TS complex in the small intestine [89]. Moreover, zein-PGA-rhamnolipid composite nanoparticles, loaded with resveratrol, have been proposed as a novel nutraceutical system for co-delivery of resveratrol and coenzyme Q10 (CoQ10), as they stabilize CoQ10 oil-in-water Pickering emulsions [90].

Thermoresponsive, lignin-based nanoparticles stabilized palm oil Pickering emulsions containing resveratrol enhance the solubility of resveratrol (33-fold of free resveratrol) as well as its light stability due to abundance in UV chromophoric groups of lignin that shield resveratrol from light degradation. Simultaneously, they offer a possibility for thermally controlled release of resveratrol, since releasing of resveratrol from emulsions decreases with elevation of environmental temperature. Resveratrol emulsions possess enhanced antioxidant activity in comparison to free resveratrol and are at the same time biocompatible, a feature that is of particular importance for their potential therapeutic use [91].

Aside from nutraceutical formulations, parenteral formulations are also explored for resveratrol delivery as a way of escaping its intestinal metabolism. Microbubbles represent innovative nanometric carriers for efficient parenteral transport of resveratrol into the cardiovascular system. They are made of spherical voids or cavities filled by gas and stabilized by a coating material such as phospholipid, surfactants, denatured human serum albumin or synthetic polymers. Ultrasound is then used to burst the microbubbles at the area of interest, thus enabling site-specific delivery [92]. Microbubbles based on acoustically active lipospheres (AALs) are an example of lipid-coated microbubbles. Resveratrol encapsulated within AALs prepared using perfluorocarbon gases and coconut oil as the cores of inner phase and with phospholipid coating showed reduced resveratrol release regardless the presence or absence of plasma in vitro, especially in the formulations containing high oil and perfluoropentane percentages that showed the slowest drug delivery rate [93].

Other hydrophilic vehicles, such as liposomes, have also been studied for use in parenteral formulations for resveratrol delivery, such as zwitterionic liposomes made from saturated phosphatidyl-choline 1,2-dipalmitoyl-sn-glycero-3-phosphocholine and cholesterol (or its positively charged derivative in cationic analog) [61] or cationic liposomes made from soya lecithin, cholesterol and stearylamine that show higher cellular uptake in vitro and even have improved biological activity in comparison to free resveratrol [94]. Liposomal resveratrol enhanced biochemical and histopathological alterations in doxorubicin-induced cardiomyopathy to a higher extent than free resveratrol. The cardioprotective effect is achieved probably through the modulatory effect on oxidative stress, inflammation and upregulated expression of cardiac S100A1 and sarco/endoplasmic reticulum calcium ATPase2a (SERCA2a) [95]. Ultra-flexible liposomes called transferosomes, consisting of phosphatidylcholine and non-ionic edge activators that enable deformability of transferosomes thus allowing their transdermal application, provide interesting perspectives for the development of resveratrol carriers. They do not affect resveratrol’s antioxidant properties while enhancing its permeability [96].

Gold nanoparticles (AuNPs) are of particular interest in biomedical application due to their physicochemical features such as controllable shape and size, as well as the potential for surface functionalization with bioactive agents [97]. Resveratrol conjugated to highly hydrophilic gold nanoparticles (AuNPs), previously functionalized with citrate and L-cysteine, maintains the biological activity of the unconjugated resveratrol [98] similar to gold nanoparticles conjugated with resveratrol via polyvinylpyrrolidone (PVP) as cross-linker [99] and gum arabic-stabilized resveratrol-encapsulated gold nanoparticles [100].

Layer-by-Layer (LbL) nanoparticles represent another innovative strategy for the delivery of resveratrol. LbL nanoparticles consisting of cationic polyallylamine hydrochloride (PAH), anionic dextran sulfate (DS) on the surface and resveratrol in the core increase resveratrol concentration in the circulation of rats following oral administration in comparison to control resveratrol in suspension. Nevertheless, resveratrol nanocores without PAH and DS coating elevated systemic concentration of resveratrol to an even higher extent due to possible destabilization of composed LbL nanoparticles upon intake [101]. For oral administration, casein-based nanoparticles loaded with resveratrol also show promise for delivering this polyphenol by increasing its bioavailability in plasma. This bioavailability was calculated to be ten times higher compared to the resveratrol administration as an oral solution [102].

The lack of appropriate delivery systems may be a somewhat limiting factor for broader clinical use of resveratrol. For delivery systems to be transformed to clinically applicable therapies, issues regarding their formulation variables, dosage and most importantly biocompatibility, need to be addressed extensively. The ratio of encapsulating material to resveratrol is an important aspect that could limit its application as a food supplement. High nanomaterial to resveratrol ratio is required to ensure resveratrol dispersibility in the aqueous-based food matrices. However, it can severely affect the organoleptic properties of orally taken formulations and the costs associated with their production [86]. Although many issues regarding their future potential therapeutic use still need to be investigated, the aforementioned delivery systems provide compelling alternatives for overcoming the low bioavailability of resveratrol and have the potential to evolve into widely applicable carriers for resveratrol.

As the present scientific literature is full of beneficial effects of resveratrol on human metabolism and pathologies, including CVD, further research regarding its cardioprotective effect and applications are encouraged. The discrepancy between clinical trials and experimental laboratory results may arise due to the poor bioavailability of resveratrol, meaning that huge concentrations of this molecule are required in food supplementation to achieve concentrations used in experimental conditions. As such, investigations regarding fabrication and application of its carriers are very attractive, with the main goal being increased water solubility and stabilization of resveratrol to increase its application potential.

While the increase of bioavailability of resveratrol may be beneficial to consumers, some adverse effects regarding its higher concentrations need to be addressed. Beneficial effects of resveratrol are correlated to its concentration and appear to be positive at low concentrations. However, when higher concentrations are reached, negative effects of this polyphenol may occur. This phenomenon is known as hormesis [103]. Concentrations of resveratrol above 10–20 µM are obtainable in vivo [38,104] and some in vitro results show that at these high concentrations, resveratrol has adverse effects, for example on endothelial cells [105,106,107]. Based on these reports, it is very important to carefully plan and monitor the doses of resveratrol since both lower and higher concentrations may fail to exert any beneficial effects.

## 3. Resveratrol and Cardiovascular Diseases

Cardiovascular diseases (CVD) are disorders which affect the heart and blood vessels [108]. There are numerous CVDs some of which are coronary heart disease (affecting vessels supplying the heart), cerebrovascular disease (affecting vessels supplying the brain), peripheral arterial disease (affecting vessels supplying legs and arms), rheumatic heart disease (Streprococcus-induced heart damage), genetic heart diseases (structural malformations), thrombosis and embolism (formation of blood clots). The most common reason for the development of acquired CVD is atherosclerosis.

Atherosclerosis is a degenerative disease characterized by the development of specific lesions called atheroma in the intima of large and medium-sized arteries. The arterial wall consists of smooth-muscle cells with variable amounts of elastic connective tissue, lined with an inner thin layer known as intima and an outer layer of collagenous connective tissue which strengthens the vessel. The intima layer consists of endothelial cells which allow diffusion of circulating fluid and cells through the endothelial surface. Atherosclerosis results from thickening of the intima due to formation of deposits of several substances, particularly lipids. Three major types of atheroma are fatty streak (accumulation of smooth-muscle cells and macrophages containing cholesterol and its esters), fibrous plaque (accumulation of extracellular lipids in the intima covered by a layer of lipid-rich smooth-muscle cells, collagen and elastin fibers) and complicated atheroma (fibrous plaque undergoing bleeding and/or calcification). It has been suggested that initial lesions in atherosclerosis are due to mechanical distortion caused by deviation of normal blood flow. The intima becomes damaged and blood platelets come in contact with connective tissue beneath releasing substance which stimulates wound healing. If this type of injury is frequent or persistent, continuous smooth-muscle proliferation occurs, connective tissue as well as lipids and calcium accumulate leading to reduced vessel lumen, obstructed blood flow, thickened intima and tissue/organ ischemia. A dangerous complication of intima thickening is a complete obstruction of blood flow and formation of blood clot causing thrombosis, pulmonary embolism and organ infarction [108,109].

Hyperlipemia and hyperlipoproteinemia are metabolism disorders in which one or more lipids and lipoproteins in blood are increased. These abnormalities can be acquired or genetically inherited. They are most often classified according to an increase in a specific lipid and/or lipoprotein. Atherosclerosis is directly correlated with an increased concentration of cholesterol, LDL and very-low-density lipoprotein (VLDL), often accompanied by a decreased clearance rate of these molecules due to an impaired function of specific receptors. On the contrary, an increased concentration of high-density lipoproteins (HDL), which promote removal and catabolism of cholesterol, has a protective effect against CVD [110].

Primary risk factors which contribute to atherosclerosis and CVD are hyperlipemia, dyslipoproteinemia, hypertension, obesity, diabetes mellitus, cigarette smoking, sedentary lifestyle and inappropriate diet. The consumption of alcohol or oral contraceptives, hypothyroidism and renal diseases were identified as less frequent risk factors [103]. Ageing is also a risk factor for developing atherosclerosis and CVD since age-related changes include reduced efficiency of metabolic processes and accumulation of unfavorable molecular species and events [111]. Several strategies have been employed to decelerate ageing and particularly vascular ageing [112,113].

### 3.1. Cardiovascular Disorders and Diabetes

It can be said that current society is in the midst of an active epidemic caused by diabetes. From 1985 to 2016, the number of people with diabetes has increased from 30 to almost 400 million [114]. CVD is a dominant cause of death in patients diagnosed with diabetes [115]. Diabetic CVD, while clinically very similar to CVD in non-diabetic people, has some significant differences. Even more, microvascular complications that accompany diabetes are unique for this condition [114].

While atherosclerosis is the major threat for microvasculature in individuals with or without diabetes, there are some specificities during its development that are distinctive for diabetes. Diabetes accelerates the development of atherosclerosis by increasing infiltration of macrophages and T lymphocytes in coronary arteries. This acceleration is followed by larger necrotic core size and more diffuse atherosclerosis [116]. Diabetes increases risk of heart failure by four-fold when all other risk factors are adjusted. Both diastolic and systolic heart failures are prominent in diabetes and contributing factors that lead to this are: cardiomyocyte dysfunction induced by diabetes, endothelial dysfunction, and increased deposition of collagen followed by fibrosis [117]. Resveratrol is able to decrease diabetes-associated fibrosis in both liver and kidneys [118]. Post-myocardial infarction (post-MI) fatality rates are nearly two times higher in diabetic patients compared to non-diabetics with the main cause of post-MI mortality being ventricular arrhythmia [119].

Dyslipidemia occurs in about 97% of diabetic patients and expresses a high correlation with atherosclerosis. Besides increased triglycerides and reduced HDL, structural changes of LDL particles are also observed in diabetes. The small, dense form of LDL is dominant in this condition [120]. This form of LDL is more atherogenic due to easier penetration and stronger attachment to the arterial wall while also being more susceptible to oxidation. What is interesting is the fact that individuals with small-size LDL particles have more of them than individuals with larger LDL, even though they may have similar LDL cholesterol levels [121]. Both oxidation and glycation of LDL occurs in diabetes. Oxidation of LDL attracts leukocytes that recognize these particles as foreign, increases the ability of leukocytes to ingest them and become foam cells, and also stimulates the proliferation of different cells, including smooth muscle cells, endothelial cells and leukocytes [122]. Glycation, on the other hand, prolongs the half-life of LDL particles while simultaneously shortening the half-life of HDL particles, thus making them less protective against atherosclerosis development [78,123]. Hypertriglyceridemia accompanies diabetes as well due to insulin-stimulated regulation of lipid flux. Insulin promotion of lipoprotein lipase together with suppression of hormone-sensitive lipase leads to reduced release of free fatty acids in the circulation [124]. Hypertriglyceridemia causes increased production of small, dense LDL, while at the same time HDL transport is negatively affected [125].

Dyslipidemia in diabetes is accompanied by endothelial dysfunction. While normal endothelium has anti-inflammatory, anti-atherogenic and vasodilatory effects, dysfunction of these processes results in acceleration of atherosclerosis. Damage of small blood vessels (and not just endothelium) can be seen throughout the body and this process is marked as microvascular damage. Local vascular dysfunction occurs due to diabetic autonomic neuropathy [126], decreased production of nitrogen oxide (NO) and increased production of endothelin-1. Low NO production and high endothelin-1 also put the vasculature in a hyper-constricted state, and stimulate increased production of pro-inflammatory cytokines such as tumor necrosis factor-α, interleukin 1β, interleukin 6, and plasminogen activator inhibitor 1. These cytokines increase vascular permeability, cause apoptosis and recruit leukocytes. Additionally, one very important consequence of inflammation is an increased production of ROS, and if the amount of produced ROS overcomes antioxidative capacity within cells, oxidative stress occurs [125]. Oxidative stress is one factor that relates all complications connected to diabetes. Diabetes is characterized as a condition with chronic oxidative stress that occurs as a result of increased metabolic flux of glucose and fatty acids [127]. Mitochondria are the main source of ROS under normal circumstances since this is the site where oxidative phosphorylation takes place. Increased ROS production occurs in mitochondria in diabetes and malfunction is considered as one of the key elements at subcellular level responsible for diabetic complications such as production of advanced-glycation end-products (AGE), insulin secretion and resistance, and oxidative stress [128]. Reduction of NO happens because of insulin resistance and hyperglycemic state [129,130]. A structural hallmark of diabetic microvascular disease is capillary basement membrane thickening, which impairs transport of metabolites and nutrients from blood to tissue and vice versa [131].

### 3.2. Thrombosis

Thrombosis is a type of CVD complication that may also occur in the setting of other diseases such as diabetes, cirrhosis, end-stage renal disease, myocardial infarction etc. Fibrinogen plays a vital role in the development of thrombosis by acquiring so-called “thrombogenic” characteristics: increased concentration, reduced porosity of formed fibrin usually with thinner and more densely packed fibrin fibrils and an increased resistance to plasmin proteolysis [132]. Fibrin formed in patients with diabetes binds tissue plasmin activator and plasminogen to a lesser extent than in healthy person, which is yet more aggravated by an increased plasmin inhibitor [133,134]. Although fibrin porosity is found reduced in certain conditions such as diabetes, its structure observed by scanning electron microscopy appears to be unaltered [135]. It can be due to the oxidation of fibrinogen as it is assumed that oxidized sites on fibrinogen are hydrophobic that results in the reduced permeability for polar components [132]. Fibrinogen is the most susceptible plasma protein to oxidation [136] and it is generally agreed that oxidation of fibrinogen has significant impact on its function [137,138,139]. By binding to small bioactive molecules that have antioxidative properties, fibrinogen can be protected from harmful oxidation, and therefore maintain its normal function [69,140]. The prevention mechanism of thrombosis by resveratrol includes its inhibitory activity on blood platelet activation, as increased activation of platelets accompanies inflammation and oxidative stress [141]. One mechanism that was found to be responsible for platelet inactivation by resveratrol was inhibition of polyphosphoinositide metabolism in activated platelets which in turn reduced the amount of these signaling molecules in platelets [142]. Another study showed that resveratrol inhibited activation of platelets in the presence of collagen by reduction of mobilization of intracellular calcium, thromboxane A2 formation, phosphoinositide breakdown, and activation of protein kinase C [141]. Not only does resveratrol inhibit platelet aggregation but it also stimulates platelet apoptosis [143]. A newer study showed that resveratrol also inhibits thrombin-induced activation of platelets by reducing mobilization of intracellular calcium. The proposed mechanism for this finding is that resveratrol acts on both phospholipase C and SOCl calcium channels in platelets [144].

### 3.3. Polyphenol-Rich Diet and CVD

There are two groups of factors that influence the development of CVD. The First group represents genetic factors that cannot be modified—gender and age—while the second group contains acquired factors those can be modified such as diet, lifestyle, environment and smoking [145].

Oxidative changes that happen during oxidative stress, which is one of the main molecular mechanisms for CVD development, can be ameliorated by antioxidants. Antioxidants are molecules capable of reacting with oxidative species, including free radicals, and by doing so they are able to prevent oxidation of other molecules (e.g., protein, DNA or fatty acid). They can be divided in two groups: enzymes (catalase, superoxide dismutase, glutathione peroxidase) and small molecules. Small antioxidant molecules can be further divided into endogenous (e.g., bilirubin and glutathione) and exogenous (e.g., ascorbic acid and polyphenols). Exogenous molecules are food constituents and can be taken either as nutrients or as food supplements. Low-grade inflammation is noted in the population with a western-type diet, while reduced levels of pro-inflammatory markers are associated with a healthy diet [146,147]. Healthy diet intervention combines multiple foods and nutrients and not just the supplementation of a single nutrient, in order to increase the range of beneficial effects. Different patterns of healthy diet have a lot of similarities; higher intake of vitamins, fibers, polyphenols, antioxidants, unsaturated fatty acids, and lower intake of saturated and trans fatty acids, refined sugar and salt [148]. Observations made in epidemiological studies suggest that consumption of foods with increased content of bioactive molecules, such as vitamins C and E, polyphenols, coenzyme Q10 and lycopene is associated with a decreased risk of atherosclerosis [149]. It is supposed that an antioxidant-rich diet is very effective in prevention of early stages of atherosclerosis by protecting LDL from oxidation and preventing endothelial oxidative lesions [150]. Here, we will focus primarily on polyphenols as food constituents and supplements with special emphasis on resveratrol and its role in prevention of CVD.

Dietary polyphenols, due to their structure, can not only scavenge free radicals, but can also chelate metal ions involved in oxidative reactions [151]. They are the most abundant antioxidants present in most plants and plant-based beverages. Food sources rich in polyphenols include fruits and vegetables, extra-virgin olive oil, red wine, coffee, black and green tea, dark chocolate, spices, nuts and seeds [152]. Many scientific reports have shown that polyphenols may delay atherosclerosis progression by several mechanisms, including antioxidant systems, reduction of adhesion molecules, attenuation of leukocyte migration and infiltration in plaque, reduction of pro-inflammatory cytokines, increase in the production of NO all of which finally leads to the reduction in blood pressure and improvement in coagulation, endothelial function and lipid metabolism [148]. One meta-analysis showed that three to five cups coffee consumption per day lowered CVD risk when compared to non-consumers [153]. Daily intake of three cups or more tea was also connected to a reduced risk of stroke [154]. Positive effects on endothelium, increase in flow-mediated dilation (non-invasive test for endothelial function), and reduction in both systolic and diastolic blood pressure were also found [155,156,157]. One study reported that green tea consumption significantly reduced LDL levels [156]. Another (cross-sectional) study concluded that reduction of inflammatory processes connected to CVD can be achieved by tea intake [158]. Cocoa powder consumption was associated with reduced production of soluble adhesion molecules sICAM-1 and sE-selectin [159]. Administration of anthocyanins led to a reduction of chemokines CXCL5, CXCL7, CXCL8 and CXCL12 [160], and significant reduction of oxidized LDL level [161]. A decrease of C-reactive protein (CRP) concentrations was related to isoflavone intake [162]. 

Contrary to the laboratory and observational studies suggesting that antioxidants might be expected to prevent CVD, an increased intake of antioxidants in human medical trials failed to exhibit any benefit in the prevention of CVD [163]. Some of those trials even reported negative outcomes [164]. There was no prevention or a delay in the development of atherosclerosis [72]. One meta-analysis that included about 300,000 individuals supplemented with vitamins A, E and C reported no preventive effects with respect to CVD [165]. While these results disappointingly suggested that CVD could not be prevented by the consumption of antioxidants, there are still some issues to be considered [163]. Firstly, it is questionable whether the correct forms of antioxidants were tested in these trials or not and whether synthetic versions of these antioxidants could completely mimic natural forms of these compounds. Secondly, antioxidants are dose and time dependent to express effects on cellular processes related to oxidative stress and CVD. Dose response was not examined, implying that some individuals were undertreated or the duration of treatment was too short for any beneficial effect to occur. Inter-individual differences in metabolism should also be considered since there may be individuals who could not benefit at all from antioxidant supplementation. For example, patients treated with statins could not exhibit any further benefit from antioxidant intake [163].

### 3.4. Effect of Resveratrol on CVD Prevention

In 1993, Fitzpatrick et al. had reported endothelium-dependent vasorelaxing activity of wine and other grape products on rat aortic rings contracted with phenylephrine that seemed to be independent of alcohol content of the wine, but rather dependent of as-yet-unknown bioactive grape skins constituents. This vasorelaxant activity appeared to be mediated by an endothelium-derived nitric oxide (eNO)-cGMP pathway and was, at least in part, ascribed to quercetin and tannic acid which in its pure form also produced endothelium-dependent relaxation, contrary to resveratrol and malvidin which did not relax the rings [166]. In addition to the vasodilatory effect achieved through enhancement of the eNO-cGMP system, resulting in cGMP-mediated vasodilation, it was then speculated that enhancement of eNO-cGMP by grape skin components could also contribute to their antithrombotic activity, since the eNO-cGMP system was previously shown to protect against platelet aggregation [167]. It was only a few years later that the cardiovascular protective effect of resveratrol was also shown and primarily been associated with its antioxidant properties, as well as enhancement of eNO production. Different experimental conditions used by Fitzpatrick et al., such as the presence of light that degrades *trans*-resveratrol, have been suggested to be the probable reason for discrepancies in the previously obtained results.

**Figure 2:**
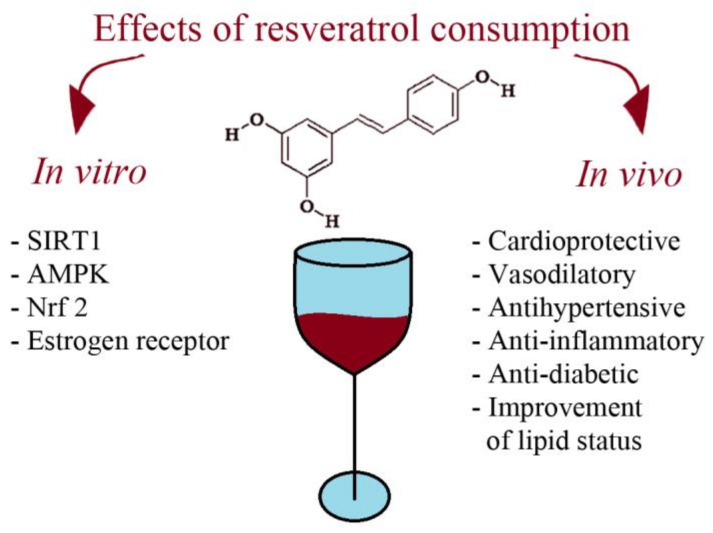
Anti-CVD effects of resveratrol found in in vitro and in vivo studies.

Several action mechanisms have been suggested to be at the root of resveratrol anti-CVD effects and some of them are presented on Figure 2.

Resveratrol interacts with around 20 molecular targets and influences the function of many molecules connected to cardiovascular disease [168,169]. It is through those direct and indirect actions that resveratrol exerts its promising potential in treating many diseases, even antioxidant activities, because resveratrol per se is considered to be a poor antioxidant, less potent than commonly administered vitamin C [170]. Resveratrol molecular targets of particular importance are the NAD^+^-dependent, class III histone deacetylase sirtuin 1 (SIRT1), the AMP-activated protein kinase (AMPK), the nuclear factor-erythroid-derived 2-related factor-2 (Nrf2) and the estrogen receptor (ER).

#### 3.4.1. Resveratrol and SIRT1

Endothelial relaxation is a mechanism that is always impaired to some extent in patients with various cardiovascular diseases. Its dysfunction as an early event in atherosclerosis is in close relationship with NO production and bioactivity, both influenced by resveratrol in a positive manner [168]. Protein deacetylase sirtuin 1 (SIRT1) can deacetlyate many histone and non-histone proteins and thus modify numerous metabolic pathways [171]. SIRT1 belongs to the conserved family of silent information regulatory genes (SIR) comprised of seven genes. SIRT1 shuttles between the nucleus and cytoplasm and is highly expressed in the endothelial cells where it can modulate angiogenic activity during vascular remodeling and growth [170,172]. It appears that SIRT1 stimulates nitric oxide synthase (eNOS) gene transcription and mRNA stability [173]. The effect of resveratrol on eNOS expression in endothelial cells is independent of the estrogen receptor [174], but is mediated by SIRT1. The knock-down of the SIRT1 gene via siRNA inhibits the resveratrol induced upregulation, but overexpression leads to the elevation of eNOS expression [175,176]. In addition, SIRT1 enhances eNOS activity: (a) SIRT1 and eNOS co-localize in endothelial cells where SIRT1 deacetylates eNOS, (b) SIRT1-induced increase in endothelial NO is mediated through lysines 496 and 506 in the calmodulin-binding domain of eNOS [172]. To further map down the RESV/STAT 1/eNOS pathway, Xia et al. [177] showed that knocking-down forkhead box protein O 1 and 3a (FOXO1 and FOXO3a) downstream of signal transducer and activator of transcription 1 (STAT 1) inhibits resveratrol induced eNOS transcriptional activation, thus suggesting that FOXO factors are involved.

**Figure 3:**
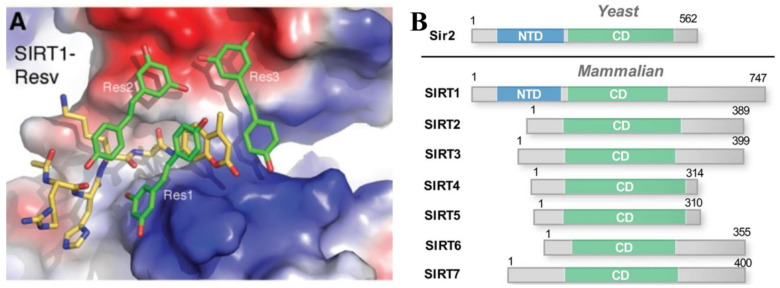
(**A**) A surface representation (with electrostatic potential distribution) of SIRT1 with the bound p53-AMC peptide and RESV. Both the peptides (carbon colored gold) and RESV (carbon colored green) are shown as a stick model. Reused with permission from Cao et al. [179]. (**B**) Comparison of yeast Sir2 amino acid sequence to seven mammalian SIRT1-7. The conserved, catalytic domain (CD) that all sirtuins have in common is colored in green. The N-terminal domains (NTDs) that are unique to yeast Sir2 and mammalian SIRT1 are colored in cyan. Numbers refer to amino acid residues in the proteins. Reused with permission from Hou et al. [180].

For a while it was under debate whether RESV has direct effect on SIRT1 because the in vitro assays with commercial fluorogenic substrates revealed that the RESV activating effects were dependent on the substrate rather than the direct interaction with SIRT1. To help in understanding Bora et al. [178] synthesized different p53 acetylpeptide substrates either lacking a fluorophore or containing a 7-amino-4-methylcoumarin (AMC) or rhodamine 110, and showed in substrate competition assays or by using crystal structures that RESV binding induced conformational changes in SIRT1 to allow for tighter binding of fluorophore carrying substrates. Structure of SIRT1 in complex with the AMC peptide revealed that two out of three bound RESV molecules mediated the interaction between the AMC peptide and the N-terminal domain (NTD) of SIRT1 (Figure 3A) thus promoting tighter binding and stimulation of SIRT 1 activity [179]. Aligning amino acid sequences of yeast sirtuin 2 (Sir2) and its mammalian homologue SIRT1 to other 6 mammalian SIRTs (2–7) shows that the NTD is unique to Sir2 and SIRT1 (Figure 3B), which may explain RESV’s selectivity toward the isoform 1 [180].

The allosteric model of RESV enhanced substrate activation of SIRT1 has some limitations, such as that it does not explain why RESV has no activity toward the native p53 substrate. A new mechanism of action was proposed based on computational MD data that highlights RESV as a stabilizer of protein-substrate interactions rather than the allosteric modulator. As proposed by the authors RESV may only restore tight binding between SIRT1 and some specific substrates which were somehow changed or mutated [180].

#### 3.4.2. Resveratrol, SIRT1 and AMPK

AMP-activated protein kinase (AMPK) is, like SIRT1, a conserved enzyme whose activity is linked to the energy status of the cell. There is a link between the AMPK and SIRT1 signaling pathways in RESV-mediated effects on the cell [181].

**Figure 4:**
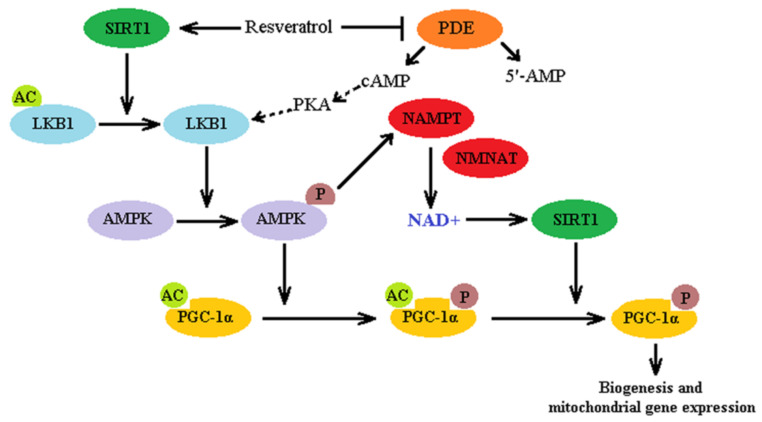
Resveratrol and putative signaling pathways involving SIRT1, AMPK and PDE. Sirtuin 1 (SIRT1), AMP-activated protein kinase (AMPK), Phosphodiesterase (PDE), cyclic AMP(cAMP), Protein kinase A (PKA), Liver kinase B1 (LKB1), PPAR gamma co-activator 1 alpha (PGC-1α).

As illustrated in Figure 4 and according to literature, RESV could in direct interaction with SIRT1 induce deacetylation of liver kinase (LKB 1), which could subsequently activate AMPK by phosphorylation [182]. RESV could also indirectly activate SIRT1 by inhibiting cAMP-degrading phosphodiesterase (PDE) in a competitive manner. Accumulation of cAMP activates Epac1, a cAMP effector protein, increases intracellular Ca^2+^ levels and activates the CamKKβ-AMPK pathway [183]. In both cases AMPK activation leads further to phosphorylation of PPAR gamma co-activator 1 alpha (PGC-1α) thus priming it for final deacetylation by SIRT1. Activated PGC-1α is an important regulator of oxidative metabolism and mitochondrial biogenesis that coactivates the nuclear respiratory factors (NRF-1 and NRF-2), which induce the transcription of genes involved in mitochondrial biogenesis [184].

As a consequence, activation of AMPK leads to an increase in the NAD^+^/NADH ratio via the nicotinamide salvation pathway (using nicotinamide phosphoribosyltransferase (NAMPT) and nicotinamide mononucleotide adenylyltransferase (NMNAT)), which in a positive loop manner activates NAD-sensitive SIRT1 [185]. AMPK activation may not always increase NAD^+^ levels, as it requires several ATPs which may not be readily available in some pathophysiological conditions [186].

#### 3.4.3. Resveratrol and Estrogen Receptor (ER)

In addition to a classical pathway where (ER) activates a signaling cascade in response to estradiol and other estrogenic compounds, ER can repress inflammatory genes through a mechanism called transrepression, via interaction with NF-κB and activator protein-1 complexes [187]. In 2013 in a screening for estrogen receptor-α (ERα) ligands which inhibit IL-6 production Srinivasan et al. reported that resveratrol was among the most efficacious [188]. Later on, the same group showed that RESV acts as an estrogen receptor-α (ERα) ligand to modulate the inflammatory response but not cell proliferation, and presented evidence to show that the anti-inflammatory activity of RESV is primarily ERα mediated [189].

**Figure 5:**
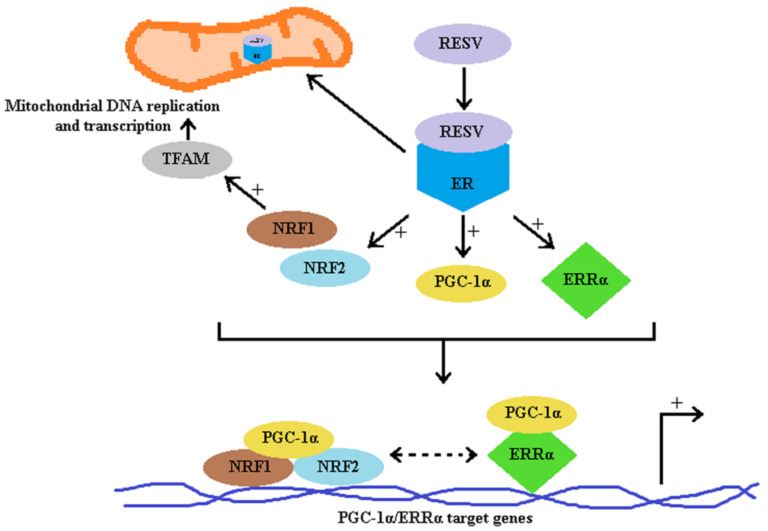
Proposed signaling pathway for the effect of resveratrol involving ER (estrogen receptor). Resveratrol (RESV), estrogen related receptor alpha (ERRα), PPAR gamma co-activator 1 alpha (PGC-1α), nuclear respiratory factors 1 and 2 (NRF1, NRF2), mitochondrial transcription factor A (TFAM).

Estrogen related receptors, named as the orphan receptors, share close structural homology to ER but do not bind natural ER ligands. There are three isoforms of ERRs, with the alpha isoform ERRα being marked as the main regulator of respiratory chain genes [190]. The effect of resveratrol is not only cell/tissue dependent but also involves different metabolic pathways. For example, in human fibroblasts, resveratrol stimulated mitochondrial functions were SIRT1 and AMPK-independent and they involved the ER and ERRα signaling pathways (Figure 5) [191].

#### 3.4.4. In Vivo Studies of Resveratrol Effect on CVD

Endothelial dysfunction, either as a cause or as a consequence, has often been considered an early marker of hypertension and atherosclerosis [192]. Endothelium-dependent vasodilatory effect of resveratrol has been shown on rat aortic rings precontracted with vasoconstrictors such as phenylephrine and KCl and has been linked to inhibition of vascular NADH/NADPH oxidase by resveratrol and the successive decrease of basal cellular O2 and NO biotransformation [193]. An antihypertensive effect of resveratrol has been reported in AngII-induced and salt-induced hypertension animal models as well as in spontaneously hypertensive rats. Activation of AMPK by resveratrol inhibits RhoA/ROCK cell signaling pathway in AngII-induced hypertensive mice, providing a possible mechanism for lowering blood pressure [194]. Reduced systolic and diastolic blood pressure, without altering heart rate, is also reported in deoxycorticosterone acetate (DOCA)-salt hypertensive mice upon resveratrol administration and is attributed to AMPK activation [195]. On the other hand, in spontaneously hypertensive rats long-term treatment with resveratrol induces reduction of systolic blood pressure through altering electrophysiological currents and Ca^2+^ signaling in sympathoadrenal chromaffin cells [196]. Resveratrol-induced reduction of systolic blood pressure by 5 mmHg was reported in clinical study involving obese humans [197]. In addition to this, high-fructose corn syrup (HFCS)-induced metabolic disturbances and hypertension in rats are ameliorated upon long-term treatment with resveratrol. Serum triglyceride, VLDL and insulin levels as well as blood pressure increase upon HFCS consumption. Impaired endothelial relaxation as well as amplified vascular contractility in response to phenylephrine and angiotensin II is associated with decreased levels of eNOS and SIRT1 and increased expression levels of gp91box and p22phox proteins, along with provoked superoxide generation in the aortic tissues of HFCS-treated rats. Coadministration of resveratrol with HFCS alleviates the harmful effect of HFCS intake on vascular function. Long-term resveratrol administration corrects triglyceride and VLDL levels, while relaxation to acetylcholine and contractions to phenylephrine and angiotensin II return to control levels and even match the values obtained in rats taking standard diet and resveratrol, probably due to promoted expression of eNOS and SIRT1 proteins in aortas from HFCS-drinking rats [198].

Increased activation of SIRT1 is also associated with anti-inflammatory effects attributed to resveratrol [199]. Given the fact that vascular inflammation underlies development of CVD, most notably atherosclerosis and hypertension, anti-inflammatory effects of resveratrol may play an important role in its cardioprotective effects [200]. Besides SIRT1 activation, anti-inflammatory effects of resveratrol have also been manifested through other anti-inflammatory mechanisms, some of which are linked to SIRT1 activation. They include down-regulation of ICAM-1 and IL-1β expression in TNF-α-stimulated human coronary arterial endothelial cells (HCAECs) [201], prevention of phenylephrine or LPS-induced up-regulation of monocyte chemoattractant protein-1 (MCP-1) in neonatal cardiomyocytes [202], inhibition of macrophage and mast cell infiltration in pressure overloaded hearts of C57BL/6 mice [203] and protection against oxidative stress and chronic inflammation in C57BL/6 mice on a high-fat diet through suppression of regulatory T-cell production and modulation of cytokines in the plasma and spleen [204]. Although some of the anti-inflammatory effects of resveratrol have been confirmed in clinical trials showing decrease in leukocyte numbers and levels of inflammatory markers in plasma following resveratrol consumption [197,205], other studies showed no significant changes in the levels of inflammatory molecules [34,206].

The results of preclinical studies are not always fully supported by clinical trials. While resveratrol has potential to interfere with many metabolic pathways which involve lipoproteins and cholesterol, its supplementation in different doses failed to show any effect on concentrations of cholesterol, LDL and HDL and triglycerides in many randomized clinical trials [207,208,209]. One study even showed a decrease of HDL cholesterol in plasma [210]. Reduction of total plasma cholesterol levels were detected only in individuals with healthy body mass index [211]. Increase of total plasma cholesterol and triglycerides was also shown in resveratrol supplementation [212]. There is a clinical study, however, that showed positive effects of resveratrol supplementation, including reduction of oxidized LDL and apolipoprotein B in plasma [213]. This study also, like previous ones, showed no alteration in LDL level, however, it is argued that both apolipoprotein B and oxidized LDL represent better markers for development of CVD [214]. It is clear that clinical results describing the effects of resveratrol on lipid profile, lipoproteins and cholesterol are contradictory, implying that more studies are needed to precisely describe the role of resveratrol (if any) on metabolism of lipids.

Resveratrol treatment of mice with pressure-overload-induced heart failure (HF) improves both cardiac and non-cardiac symptoms of HF. Reduced cardiac fibrosis, improved diastolic function, down-regulation of several HF-characteristic hypertrophy and extracellular matrix remodeling markers, restored levels of mitochondrial oxidative phosphorylation complexes as well as restored AMP-activated protein kinase activation were observed in resveratrol-treated mice. Non-cardiac symptoms such as peripheral insulin sensitivity and vascular function were also improved [215]. Besides rodents, resveratrol improves insulin sensitivity and glucose metabolism in non-human primates, as well [216]. Reduction of oxidative stress, improved uptake of glucose, its reduced production in hepatocytes and activation of AMPK are all associated with positive effects of resveratrol on glucose metabolism [217]. Protective effects of resveratrol against diabetic complications including improved insulin resistance, hyperglycemia and hyperinsulinemia are also shown in some clinical trials [217,218,219]. One meta-analysis has shown that short term resveratrol supplementation reduces insulin resistance, insulin concentration and fasting glucose in patients with diabetes [219]. Another study showed no measurable effects in type 2 diabetic patients after six months long resveratrol supplementation. However, the authors of the study do not exclude the possibility that certain groups of patients, such as those with shorter diabetes duration, might benefit from resveratrol supplementation [212].

Despite the remarkable accumulation of evidence obtained in abovementioned in vitro studies supporting beneficial effects of resveratrol, it can be noted that in vivo results don’t correlate completely to those obtained in in vitro experiments. An important aspect to consider is the concentration of resveratrol used in in vitro experiments that is often 10–100 times greater than the one observed in human plasma after oral consumption [220]. In addition to shortcomings related to inherent biological limitations of resveratrol, gut microbiota and genetic background account for inter-individual responses to orally administered resveratrol [98]. Given the non-physiological concentrations used in in vitro studies and omission of the contribution of resveratrol metabolites to measured effects, translation of results obtained in in vitro experiments to those obtained in clinical trials is somewhat difficult.

## 4. Resveratrol and CVD Related to COVID-19

During 2020, many meta-analyses and meta-meta-analyses reported the positive association between underlying diseases, especially CVD, and the illness severity among COVID-19 patients [221,222,223,224,225]. Resveratrol can alleviate the numerous factors associated with CVD, so it has potential as a functional supplement to reduce COVID-19 illness severity in patients displaying poor prognosis. Resveratrol was shown to mitigate the major pathways involved in the pathogenesis of SARS-CoV-2 including regulation of the renin-angiotensin system and expression of angiotensin-converting enzyme 2 (ACE2), stimulation of immune system and downregulation of pro-inflammatory cytokines release [226]. Therefore, several studies already have anticipated potential implementation of resveratrol in COVID-19 treatment.

As resveratrol is known for its antiviral effects against number of the respiratory tract viruses [227] including MERS-CoV [228], its antiviral potential against SARS-CoV-2 was investigated, which demonstrated that resveratrol inhibits the replication of SARS-CoV-2 in cultured Vero cells, and as a result, its potential utility as a novel therapy was proposed [229]. In addition, resveratrol is also recognized as cardioprotective supplement for mitigation of cardiotoxicity associated with chloroquine/hydroxychloroquine treatment in SARS-CoV-2 patients reinforcing its antiviral potency [230]. The study based on molecular dynamic simulation revealed highly stable bound conformation of resveratrol to SARS-CoV-2 spike protein: ACE2 receptor complex, resveratrol and other stilbene-based natural compounds were recognized as promising candidates for development of drugs against COVID-19 [231].

COVID-19 patients develop a pro-coagulative state and thrombotic lesions in their pulmonary microvessels have a twice as much higher prevalence than in non-COVID-19 critical patients [232]. The hypercoagulation and thromboembolic complications correlate with a more severe course of COVID-19 and high thromboembolism risk of COVID-19 has shown to be directly associated with a higher risk of mortality according to a systematic review and meta-analysis [233]. With its proven anti-thrombotic effects, resveratrol was proposed as an adjunct treatment for slowing and ameliorating vascular thrombosis in the course of COVID-19 [234].

Hyperinflammatory syndrome induced by SARS-CoV-2, resulting from a dysregulated host innate immune response, most likely leads to disease severity and mortality in COVID-19 [235]. Anti-inflammatory properties of resveratrol have been vastly studied on animal models, cell lines and human subjects in which resveratrol demonstrated reduced inflammatory cell production and pro-inflammatory cytokine accumulation [236]. Therefore, resveratrol could be beneficial for modulation of inflammation without compromising the adaptive immune response. Indeed, as novel approach to prevent COVID-19-induced cytokine storm, nutraceutical agents targeting inflammation-modulating microRNAs were proposed, with resveratrol being one of them [237].

In addition to mentioned beneficial effects, sanatory effect of resveratrol involved in the pathogenesis of SARS-CoV-2 related to CVD should also be considered. SARS-CoV-2 enters the cells via binding to ACE2 expressed on cells and virus internalization may cause a downregulation of ACE2 on host cell surface. This leads to a localized boost of angiotensin II (AII) level accompanied with a reduced angiotensin 1-7 (A1-7) level, resulting in imbalance between these angiotensins, which finally causes lung and heart damage [238]. Resveratrol was found to suppress angiotensin II (Ang II)/angiotensin II type 1 receptor (Ang II/AT1R) axis and enhance angiotensin 1-7/Mas receptor (Ang 1-7/MasR) axis [239], and resveratrol protection against arterial aging was shown to be associated with stimulation of Ang 1-7/MasR axis [240]. Therefore, resveratrol could be a suitable supplement to mitigate the imbalance of renin-angiotensin system that has pivotal role in the pathogenesis of COVID-19.

Vascular endothelial cells can be infected by SARS-CoV-2 as ACE2 is expressed abundantly on these cells, and accordingly widespread endothelial injury and inflammation has been observed in advanced COVID-19 cases [241]. Resveratrol is known to enhance endothelial NO production by upregulation of eNOS expression, increasing its activity. Also, resveratrol is observed to reduce endothelial oxidative stress and endothelin-1 synthesis [242]. Accordingly, resveratrol can have protective effects on vascular function, thus alleviating end-organ damage and thrombotic events in severe COVID-19 cases.

In COVID-19 patients with severe diseases, immune cell cytotoxicity is impaired with decreased numbers of circulating T, B, and NK cells and skewed CD8^+^ T cells toward a terminally differentiated/senescent phenotype [243]. Resveratrol regulates immunity by interfering with immune cell regulation via activation of macrophage, cytotoxic T cell and natural killer (NK), as well as in CD4^+^CD25^+^ regulatory T cell suppression [244]. Hence, resveratrol could have contributed to restoration of the cytotoxic potential of immune cells, and thus n clearance of SARS-CoV-2 infection. It could also aid in restoring control of immune responses, thus ameliorating tissue/organ damage.

During COVID-19 infection, the systemic oxidative stress is a consequence of multiple biological activities. However, oxidative stress is also related to viral interaction with ACE2, where disulfide-thiol and NADPH/NADP+ balance affect oxidation state of ACE2, and that is why redox-modulating agents were proposed in the treatment of infection [245]. With its extraordinary antioxidant potential and ability to inhibit oxidative stress [246], resveratrol could have significant beneficial effects not only to mitigate COVID-19-induced tissue/organ damages, but also it could contribute in preventing viral protein binding on the host cells.

**Figure 6:**
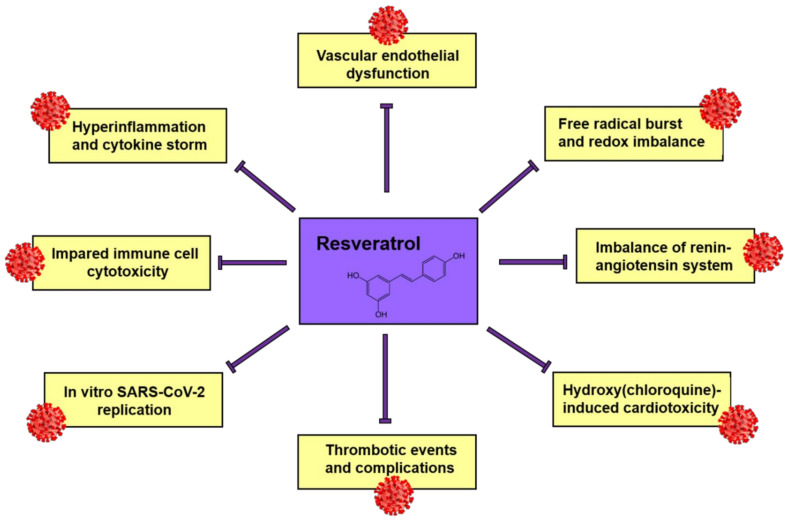
Potential of resveratrol in mitigation of COVID-19 infection severity related to CVD.

Finally, resveratrol could alleviate the course of COVID-19 disease, reduce its consequences and perhaps decrease morbidity risk by simultaneous varied desirable effects during COVID-19 infection as well as convalescence. Resveratrol potential in mitigation of COVID19 infection severity related to CVD is summarized in Figure 6.

Additional to these pharmacological benefits, resveratrol is a naturally occurring compound that is easily accessible to the health-oriented consumers in affordable costs with safe oral intake doses (studies on dose toleration). For these reasons, recent clinical investigations of resveratrol application in combination with essential minerals for COVID-19 therapy have already begun. A trend towards reduction in mortality was observed in patients consuming combination of resveratrol (5.6 mg) and copper (560 ng) [247]. Currently, resveratrol-assisted zinc therapy (Reszinate) is also under investigation for reduction of SARS-CoV-2 viral load and COVID-19 severity (ClinicalTrials.gov: NCT 04542993).

## 5. Conclusions

Solid scientific evidence on the beneficial effects of resveratrol in prevention and control of CVD has for long been an excuse for wine lovers to get yet another glass of wine: Mediterranean diet despite high intake of saturated fat and moderate intake of wine does not predispose for cardiovascular disorders. Even though matter of dispute on the exact mode of action, resveratrol rich Mediterranean diet provides protection from CVD at many different levels. Data gathered from in vitro studies indeed confirmed specific molecular targets of resveratrol action that could directly be attributed to its role in prevention and control of CVD. Not surprisingly, resveratrol supplements are popular among health-oriented consumers. Of the diseases particularly relevant to be further explored in relation to resveratrol action is COVID-19 disease, due to frequent occurrence of cardiovascular complications in severely ill patients. Living in a pandemic for which no effective drugs exist, nor an easily available vaccine, has caused us to turn more to naturally occurring remedies that may boost immune system, and ameliorate systemic effects of the disease, in particular, life threatening complications of cardiovascular system. For these reasons, clinical investigations of resveratrol in combination with essential minerals such as zinc and copper for COVID-19 therapy have already begun. Improvements in the delivery strategies for resveratrol that can stabilize its structure and improve its stability have already been made with innovative delivery vehicles. In conclusion, regular intake of resveratrol rich diet, or resveratrol-based complementary medicaments, may contribute to a healthier cardiovascular system, and prevention and control of cardiovascular disorders.

## Figures and Tables

**Table 1:** Abundance of resveratrol in different sources. Data was obtained from Phenol-Explore database (http://phenolexplorer.eu/, accessed on 15 February 2021).

Source	Amount of Resveratrol
Red grape wine	0.27 mg/100 mL
Rose grape wine	0.12 mg/100 mL
White grape wine	0.04 mg/100 mL
Muscadine grape red wine	1.41–4.41 mg/100 mL
Lingonberry	3 mg/100 g FW
Cranberry	1.92 mg/100 g FW
Redcurrant	1.57 mg/100 g FW
Bilberry	0.67 mg/100 g FW
Strawberry	0.35 mg/100 g FW
Black grapes	0.15 mg/100 g FW
Green grapes	0.02 mg/100 g FW
Dark chocolate	0.04 g/100 g
Pistachio	0.11 mg/100 g
Peanut	0.04 mg/100 g
